# Ultra-efficient frequency comb generation in AlGaAs-on-insulator microresonators

**DOI:** 10.1038/s41467-020-15005-5

**Published:** 2020-03-12

**Authors:** Lin Chang, Weiqiang Xie, Haowen Shu, Qi-Fan Yang, Boqiang Shen, Andreas Boes, Jon D. Peters, Warren Jin, Chao Xiang, Songtao Liu, Gregory Moille, Su-Peng Yu, Xingjun Wang, Kartik Srinivasan, Scott B. Papp, Kerry Vahala, John E. Bowers

**Affiliations:** 1 Department of Electrical and Computer Engineering, University of California, Santa Barbara, CA 93106 USA; 2 State Key Laboratory of Advanced Optical Communications System and Networks, Peking University, Beijing, 100871 China; 3 T. J. Watson Laboratory of Applied Physics, California Institute of Technology, Pasadena, CA 91125 USA; 4 School of Engineering, RMIT University, Melbourne, VIC 3000 Australia; 5 Microsystems and Nanotechnology Division, National Institute of Standards and Technology, Gaithersburg, MD 20899 USA; 6 Time and Frequency Division, National Institute of Standards and Technology, Boulder, CO 80305 USA

**Keywords:** Microresonators, Nonlinear optics, Frequency combs

## Abstract

Recent advances in nonlinear optics have revolutionized integrated photonics, providing on-chip solutions to a wide range of new applications. Currently, state of the art integrated nonlinear photonic devices are mainly based on dielectric material platforms, such as Si_3_N_4_ and SiO_2_. While semiconductor materials feature much higher nonlinear coefficients and convenience in active integration, they have suffered from high waveguide losses that prevent the realization of efficient nonlinear processes on-chip. Here, we challenge this status quo and demonstrate a low loss AlGaAs-on-insulator platform with anomalous dispersion and quality (*Q*) factors beyond 1.5 × 10^6^. Such a high quality factor, combined with high nonlinear coefficient and small mode volume, enabled us to demonstrate a Kerr frequency comb threshold of only ∼36 µW in a resonator with a 1 THz free spectral range, ∼100 times lower compared to that in previous semiconductor platforms. Moreover, combs with broad spans (>250 nm) have been generated with a pump power of ∼300 µW, which is lower than the threshold power of state-of the-art dielectric micro combs. A soliton-step transition has also been observed for the first time in an AlGaAs resonator.

## Abstract (web summary)

Despite larger nonlinear coefficients, waveguide losses have prevented using semiconductors instead of dielectric materials for on-chip frequency-comb sources. By significantly reducing waveguide loss, ultra-low-threshold Kerr comb generation is demonstrated in a high-*Q* AlGaAs-on-insulator microresonator system.

## Introduction

There has been extensive research on integrated nonlinear photonics in the last few years, driven by breakthroughs in microcombs and other on-chip nonlinear devices. This has opened up many new opportunities for on-chip integrated photonics, ranging from spectroscopy to atomic clock applications^1–3^. The need for efficient nonlinear devices has motivated the development of different material platforms in nonlinear photonics. A common goal of these efforts is the reduction of the waveguide propagation loss to enable high-*Q* cavities. These are used to resonantly enhance power and therefore increase the efficiency of the nonlinear optical processes^4^. In this regard, silica on silicon resonators^5–7^ have long been dominant offering *Q* factors of nearly 1 billion^6^. These devices can access a wide range of nonlinear effects including microwave rate soliton microcombs^8^. However, over the last 5 years, there has been remarkable progress to significantly improve the *Q* factors of resonators in many other nonlinear integrated optical material platforms. One example is the Si_3_N_4_ platform, which delivers high performance in Kerr comb generation on-chip^9–11^. Si_3_N_4_ microresonators have enabled the generation of efficient frequency combs with repetition rates from microwave to THz frequencies^12^ and feature *Q* factors beyond 30 million^13,14^. Similarly, LiNbO_3_ offers additional opportunities for integrated nonlinear devices, due to its strong second order nonlinearity and electro-optic efficiency^15^. The significant reduction of waveguide loss in the lithium niobate on insulator (LNOI) platform^16,17^ enabled the demonstration of resonators with *Q* factors beyond 10 million^18^. And its combined third and second order nonlinearities have also allowed soliton microcomb formation with intrinsic second-harmonic generation^19^.

However, despite significant progress, there remain many challenges in the current technologies of integrated nonlinear photonics. One problem is efficiency as measured by threshold or turn-on power. Focusing on Kerr-effect microcombs, all of the dielectrics noted above feature weak nonlinear Kerr indices (*n*_2_ is usually in the 10^−19^ m^2^ W^−1^ range or less). To reduce turn-on power and comb operation power to levels accessible by integrated lasers (tens of mW)^20^, there are primarily two approaches in use. The first is to boost optical *Q* factor, which provides a quadratic benefit to lowering the Kerr-effect parametric threshold^21^. However, to obtain high quality factors, the material absorption and waveguide scattering need to be carefully suppressed during microfabrication. A second approach is to reduce the mode volume of the resonator in order to decrease the required stored optical energy at threshold. Here, however, simply reducing the diameter of the resonator is not always an option as certain applications require large resonator diameters so as to obtain electronic-rate microcombs. Therefore, the reduction of the modal cross sectional area is also used to improve efficiency^14^. And while this approach has been particularly fruitful in silicon nitride microcombs, it does increase the challenge and complexity of maintaining high optical *Q* factor in the highly confined waveguides. As a result, it is common to have a large number of additional fabrication steps for high quality resonators^12,14^. These include chemical-mechanical polishing (CMP) and high temperature anneals. These additional steps can complicate the fabrication process, increase the cost, and sacrifice the yield and precise control of geometry, all of which add difficulties for high volume, low cost production in industry. A final and significant point is that the above dielectric nonlinear microcavities also encounter difficulties when they are integrated with active components due to incompatibilities in material, design and fabrication, thereby hindering the realization of fully integrated nonlinear photonic circuits.

One way to address these problems is to use III–V semiconductor materials^22–25^. III–Vs feature extremely large nonlinear coefficients that are typically orders-of-magnitude larger than those of the dielectric materials noted above^26^. Also, their refractive indices are higher, which can be used to achieve a high index contrast and therefore a small modal cross sectional area (high intensity). These two factors are very attractive as they reduce the turn-on power of nonlinear optical processes in semiconductor-based nonlinear optical platforms. Moreover, III–V materials feature both a second- and a third-order nonlinear coefficient, both of which are required in frequency-comb systems^27^. Furthermore, the wide usage of semiconductors in integrated photonics^20^, as passive and active (laser and detector) elements, provides the potential for the integration of nonlinear devices into PICs.

However, most of the III–V semiconductor photonic platforms suffer from high waveguide losses, usually in the order of several dB cm^−1^^28–30^, which correspond to quality factors of ∼10^5^ or lower. The lack of sufficiently high *Q* in semiconductor platforms has limited the ability to harness their attractive material properties for nonlinear optics applications like microcombs. Recent demonstrations of whispering gallery mode or partially etched waveguide cavities in (Al)GaAs^31,32^ achieved quality factor in the millions. However, significant challenges remain to obtain such high-*Q* factors within a waveguide that is both suitable for nonlinear applications such as Kerr microcombs or second-harmonic generation (SHG) while also being compatible with integration. For example, fully dry etched surfaces are required in these structures as opposed to partially etched waveguides.

In this work, we make a key step in this direction by demonstrating compact micro-ring resonators in the AlGaAs-on-insulator (AlGaAsOI) platform with an intrinsic quality factor beyond 1.5 × 10^6^. The waveguides are fully etched with sub-micron dimensions and exhibit anomalous dispersion at a wavelength of ∼1.55 µm. Their high quality factor, high Kerr nonlinear coefficients and compact mode volume, enabled us to demonstrate ultra-efficient frequency-comb generation. For a 1 THz comb, the threshold power is only ∼36 µW, which is a 100 times reduction relative to previous semiconductor resonators and 10 times lower compared with the state-of-the-art results from integrated dielectric microresonators. With only 300 µW pump power we were able to generate a frequency comb that covers a spectral range of ∼250 nm. A soliton-step transition has also be observed. Furthermore, the waveguide platform features much simpler fabrication procedures compared with previous high-*Q* nonlinear platforms, and thus is suitable for high volume, low cost production. This demonstration paves the way to ultra-efficient nonlinear photonics of semiconductors, and provides a valuable solution to nonlinear PICs in the near future.

## Results

### Device design

The nonlinear optical platform used in this work is AlGaAsOI. One key reason for selecting AlGaAs as a nonlinear material is its relatively large bandgap compared with other commonly used semiconductors in photonics, such as Si (1.1 eV (1127 nm)) or InP (1.34 eV (925 nm)). By changing the Al mole fraction, the bandgap of Al_x_Ga_1−x_As varies from 1.42 eV (872 nm) to 2.16 eV (574 nm)^33^, which can be harnessed to avoid two photon absorption (TPA) at the two most important telecom bands (1310 and 1550 nm). In this work, we chose *x* to be 0.2 for operating the comb at C-band wavelengths. Higher Al portion can be used when targeting shorter pump wavelengths. Furthermore, AlGaAs has a very high nonlinear optical coefficient *n*_2_ = 2.6 × 10^−17^ m^2^ W^−1^, which makes it a very attractive in terms of nonlinear efficiency.

**Fig. 1:**
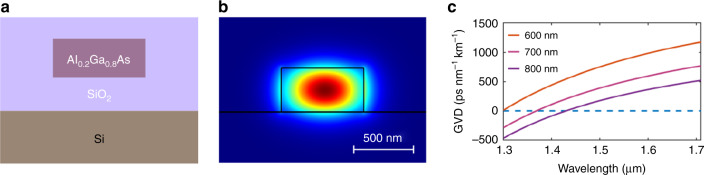
Waveguide design and dispersion engineering. **a** Schematic drawing of the AlGaAsOI waveguide cross section; **b** simulated intensity distribution of the waveguide fundamental TE mode for comb generation; **c** simulated GVD of 400-nm-thick AlGaAsOI waveguides with different widths.

A schematic drawing of the fully etched AlGaAsOI waveguide cross section is shown in Fig. 1a. One essential requirement for Kerr comb generation is that the waveguide should have anomalous group velocity dispersion (GVD)^3^ at the pump wavelength. Our simulations indicated that the AlGaAsOI waveguide needs to have sub-micron dimensions in order to obtain such dispersion at the two widely used telecom bands (O band and C band). This is due to the strong material dispersion of Al_0.2_Ga_0.8_As, which needs to be compensated by dispersion engineering of the waveguide geometry. In this work, the AlGaAs layer thickness is set to be 400 nm, at which the calculated GVD is anomalous at C-band wavelengths for waveguides with several different widths, as shown in Fig. 1c. The simulated mode distribution of one example waveguide (400 nm × 700 nm) is plotted in Fig. 1b. Compared with the commonly used Si_3_N_4_ waveguides for comb generation, the mode volume is reduced by a factor of ∼4, which not only enhances the photon intensity, but also enables more compact devices.

The fabrication of the AlGaAsOI platform is based on heterogeneous wafer bonding technology, similar to the previous process of GaAsOI devices used for second-harmonic generation^34^, and is discussed in detail in the Methods section.

### Waveguide loss reduction

The propagation loss of the waveguides needs to be low in order to achieve high-*Q* resonators. This requires the reduction of the sidewall roughness, which plays a major role in limiting the loss of the AlGaAsOI platform. There are two reasons for this: first, the mode size of the waveguide is small, which leads to a significant interaction of the mode with the waveguide sidewall; second, the strong index contrast between AlGaAs and SiO_2_ causes increased scattering loss, which scales with (${{n}^{2}_{\mathrm{core}}}-{{n}^{2}_{\mathrm{clad}}}$)^35^. As a result, it is critical to lower the sidewall and surface roughness in all high index contrast platforms in order to obtain low propagation loss.

**Fig. 2:**
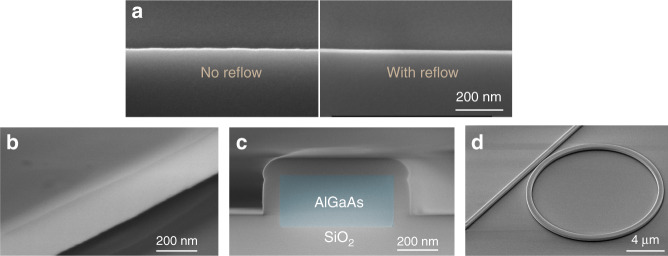
SEM images. **a** Top view of the SiO_2_ hard masks with and without reflow applied after the lithography process. **b** Sidewall of the waveguides. **c** Cross section of the waveguide after passivation and a thin layer of SiO_2_ deposition; the AlGaAs core is highlighted with false color (blue). **d** A ring resonator.

In this work we reduced the scattering loss mainly by two means. The first one is the implementation of a reflow process of the patterned photoresist after the lithography process. Figure 2a shows the SEM top-view images of the patterned SiO_2_ hardmask with and without reflow of photoresist, both of which are exposed using an ASML 248 nm DUV stepper. It can be seen that without reflow, a significant amount of roughness is visible at the edge of the SiO_2_ hardmask. This would be transferred to the sidewall of waveguide after AlGaAs etch and would act as a strong optical scattering source. When a resist reflow step is applied after the lithography process, the resist boundary is smoothed out and the roughness is hardly visible in the SEM image. The change of resist shape caused by reflow can be calibrated and taken into account in mask design, which enables dimensional control of the waveguide width with nanometer scale.

The second means to reduce the scattering loss is an optimized dry etch process. Here we applied an Inductively Coupled Plasma Etching (ICP) etch for both the hardmask and the AlGaAs. The gases used are CHF_3_/CF_4_/O_2_ for etching the SiO_2_ and Cl_2_/N_2_ for etching the AlGaAs. The well-developed etching recipes resulted in a smooth etching profile as shown in Fig. 2b. The SEM picture of the cross section of the waveguide (Fig. 2c) shows that the sidewall angle is very close to 90°, which is beneficial for accurate geometry control.

Besides the scattering, another loss contributor for semiconductor waveguides is the absorption caused by defect states at material surfaces. Recent research shows that this factor starts to become important for resonators operating in the high-*Q* region^31^. A way to reduce loss originating from this source is to apply a surface passivation treatment to the waveguide, which can eliminate the intra-band states. In this work, a 5-nm-thick Al_2_O_3_ layer was deposited by atomic layer deposition (ALD), which fully surrounds the waveguide core and passivates the AlGaAs surface. Other methods to reduce the surface dissipation, such as wet nitridation, have also been discussed in the literature^31^ and may further improve the passivation.

### Characterizations of microresonators

As indicated above, to generate a frequency comb in ring resonators, the dispersion of the waveguide plays a critical role. To characterize the GVD of the waveguides, we fabricated a ring resonator with 100 µm radius and a free spectral range (FSR) of 118 GHz. Using this resonator we measure the resonance frequency of a mode family as a function of relative mode number *µ*, relative to a reference resonance at *ω*_0_, which is around 1550 nm. The resonance frequency *ω*_*µ*_ of the modes can be expanded in Taylor series as:

1 $$\omega _\mu = \omega _{\mathrm{0}} + \mu D_{\mathrm{1}} + \,\frac{1}{2}\mu ^{\mathrm{2}}D_{\mathrm{2}} + \,\frac{1}{6}\mu ^{\mathrm{3}}D_{\mathrm{3}} + \cdots$$

**Fig. 3:**
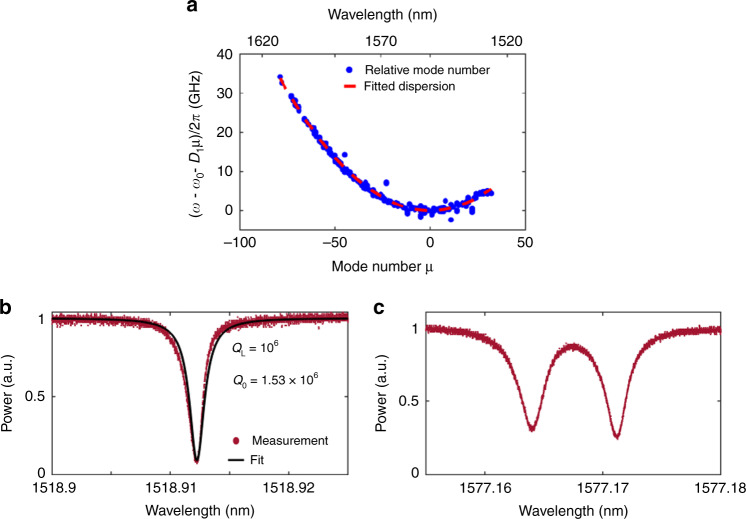
Linear characterization of the resonator. **a** Measured relative mode frequencies *D*_int_ plotted versus *µ*. **b** Measured transmission spectrum of a resonance around 1518 nm (red dots) and fitting curve (black line). **c** Resonance with splitting due to backscattering (red dots).

Where *D*_1_/2π refers to the FSR around *ω*_0_ and *D*_2_ is related to the GVD *β*_2_ by $D_2 = - \frac{c}{n}D_1^2\beta _2$. Figure 3a shows the measured relative mode frequencies $D_{{\mathrm{int}}} \equiv \omega _\mu - \omega _0 - \mu D_1$. By fitting the data, second order dispersion *D*_2_/2π is extracted to be 10.8 MHz. This confirms that the resonator is operating in the anomalous dispersion regime.

The quality factor of the resonators is estimated based on the transmission spectrum of the resonances. Figure 3b shows a resonance at a wavelength of ∼1519 nm for a 1 THz ring resonator, which has waveguide width of 700 nm and radius of 12 µm. The intrinsic quality factor is measured  to be ∼1.53 × 10^6^, which corresponds to a propagation loss around 0.4 dB cm^−1^. The *Q* factor is one order of magnitude higher compared with that of previous AlGaAsOI resonators for comb generation with a similar radius^36^. Considering the compact mode size (∼0.28 µm^2^) and small radius of the ring, it is reasonable to believe that this platform has waveguide losses that are comparable to state-of-the-art fully etched silicon on insulator (SOI) or even many commonly used dielectric waveguides.

Figure 3c presents the transmission spectrum of a resonance, which shows a splitting of the mode. This phenomenon is usually observed in high-*Q* resonators^37^, which are sensitive to small imperfections or scatterers at waveguide surfaces. This indicates that the quality factors are still limited by the scattering loss and can be further reduced by optimizing the fabrication process.

**Fig. 4:**
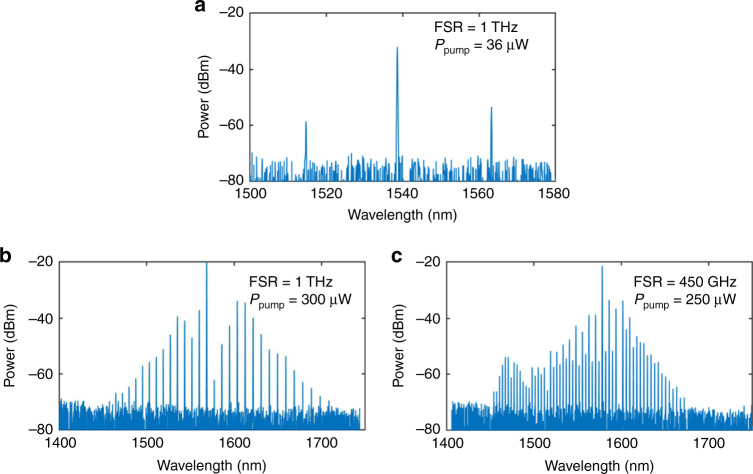
Frequency-comb characterization. Spectrum generated by a 1 THz resonator under pump power of (**a**) 36 µW and (**b**) 300 µW, and a 450 GHz resonator under pump power of (**c**) 250 µW.

Next we investigate the nonlinear optical efficiency of the waveguides to illustrate the advantage of combining high nonlinear coefficients, high index contrast and high quality factors simultaneously in this AlGaAsOI platform. Figure 4a shows the parametric oscillation spectrum of such a 1 THz resonator operated at 36 µW, where one can observe the onset of frequency-comb generation. This indicates that we were able to generate efficient Kerr combs by pumping the resonators at C-band wavelengths. The threshold power is ∼100 times lower compared with previous AlGaAsOI resonators with similar FSR^36^ and is consistent with the fact that the *Q* factor has a 10 times improvement.

Besides the significant reduction of the threshold power, the high *n*_2_ of AlGaAs highlights its advantages even more dramatically in terms of the efficiency of comb broadening above the threshold. It can be seen in Fig. 4b that for the same 1 THz resonator discussed above, the generated comb lines cover a >250 nm wide spectral range under a power of only 300 µW. For another comb with 450 GHz FSR, 250 µW pump power enables a comb span over 200 nm with >50 comb lines. Note that these pump powers are lower than the record threshold power reported for Si_3_N_4_ microcombs^14^. Using such quality factor, only a few mW pump power should be sufficient to generate an octave span THz comb with a double dispersive wave if proper dispersion engineering is applied, which is essential for *f-2f* self-referencing and frequency synthesis applications^1^.

## Discussion

A comparison of different nonlinear material platforms for frequency-comb generation is shown in Table 1. The demonstration in this work, to the best of our knowledge, delivers the lowest threshold power for comb generation so far. It needs to be noted that the Si_3_N_4_^14^ and silica^7,21,38^ platforms with the highest *Q* could potentially have a similar threshold when implemented as a THz comb, if it is assumed that their high *Q* at the smaller FSR (larger resonator) can be maintained at the larger FSR (smaller resonator). This, however, is challenging in practice, because smaller ring radii typically feature increased waveguide loss either due to enhanced bending or scattering loss^21,39^. On the other hand, this scaling of loss with radius also suggests that the *Q* factor of the present AlGaAsOI resonator can be even higher for larger rings.

**Table 1:** Performances of various nonlinear materials for microcomb generation.

Material	Refractive index	*n*_2_ (m^2^ W^−1^)	Mode area (µm^2^)	Highest *Q* for comb generation	Lowest threshold power (mW) (FSR)
Silica^38^ (Wedge disk)	1.45	3 × 10^−20^	∼60	6.7 × 10^8^	1.2 (9.3 GHz)
Silica^21^ (Microtoroid)			∼10	1.2 × 10^8^	0.17 (1 THz)
Si_3_N_4_^14^	2.0	2.5 × 10^−19^	∼1.5	3.7 × 10^7^	0.33 (200 GHz)
LiNbO_3_^19^	2.21	1.8 × 10^−19^	∼1	2.2 × 10^6^	4.2 (199.7 GHz)
Ta_2_O_5_^50^	2.05	6.2 × 10^−19^	∼1.5	3.2 × 10^6^	10 (500 GHz)
Hydex^51^	1.7	1.15 × 10^−19^	∼2	1 × 10^6^	50 (200 GHz)
Si^52^	3.47	5 × 10^−18^	∼2	5.9 × 10^5^	3.1 (127 GHz)
GaP^53^	3.05	6 × 10^−18^	∼0.15	3 × 10^5^	10 (500 GHz)
AlN^54^	2.12	2.3 × 10^−19^	∼1	9.3 × 10^5^	NA
AlGaAs (this work)	3.3	2.6 × 10^−17^	∼0.28	1.5 × 10^6^	0.036 (1 THz)

The key advantage offered by using AlGaAs is its high Kerr nonlinear coefficient (*n*_2_ = 2.6 × 10^−13^ cm^2^ W^−1^), which is two orders-of magnitude higher than that of Si_3_N_4_ (*n*_2_ = 2.5 × 10^−15^ cm^2^ W^−1^). This is apparent by considering the expression for the parametric oscillation threshold power (*P*_th_*)* of a comb^21,40^:

2 $$P_{{\mathrm{th}}} \approx 1.54\frac{\pi }{2}\frac{1}{\eta }\frac{n}{{n_{\mathrm{2}}}}\frac{\omega }{{D_{\mathrm{1}}}}\frac{A}{{Q_{\mathrm{T}}^{\mathrm{2}}}}$$

where *η* *=* *κ*_e_*/κ* is the coupling factor (*κ* and *κ*_e_ are total and cavity related decay rate), *n*_2_ (*n*) refers to the Kerr nonlinear index (refractive index), *A* is the mode area and *Q*_T_ refers to the total quality factor of the resonator. To highlight the importance of the highly nonlinear optical material and small mode volume, we consider an example comparing the influence of two different material systems on the threshold of comb power. For this exercise we initally assume that the resonators in the different materials are operated at the same frequency with the same FSR, and have the same waveguide coupling parameter and total Q factor. In this case, only the nonlinear coefficient, mode area, and the refractive index of the waveguide determine the threshold power. For AlGaAsOI waveguides (*n* = 2.9, *A* = 0.28 µm^2^), the threshold power for the corresponding AlGaAsOI resonator will be ∼270 times lower compared with that of a  Si_3_N_4_ standard microresonator design. Alternatively, to achieve the same threshold power in both systems, the quality factor required for the AlGaAsOI resonator can be 16 times lower than for the state-of-the-art Si_3_N_4_ technology. Moreover, the reduced Q factor  relaxes the strict fabrication requirements to achieve low loss waveguides .

Another advantage offered by AlGaAs or other epitaxial grown materials is precise thickness control. Most of the dielectric thin films, either prepared by direct deposition or smart-cut bonding technology, usually require chemical-mechanical polishing (CMP) to reduce their surface roughness and thereby boost optical *Q* factor. This process removes a significant amount of material and introduces a non-uniformity to the film thickness on the order of tens of nanometer over the wafer scale. As a result, such ultra-high-*Q* resonators can suffer from significant waveguide geometry variation, which is problematic in many applications that rely upon accurate control of dimensions. For example, the detection of carrier envelope offset frequency of microcombs for self-referencing or the efficient spectral translation between visible and infrared light require precise dimensional control of the waveguide cross section^41^. Compared with dielectrics, the epitaxial growth for semiconductors enables atomic scale smooth surfaces (see Supplementary Fig. 1) and accuracy of material thickness with high uniformity, providing a good solution to dimensional control in nonlinear photonics.

One commonly used step in the fabrication of high-*Q* resonators, but not critical in AlGaAsOI process, is a high temperature (>1000 °C) anneal. This is needed to reduce the O–H and N–H bonds in deposited Si_3_N_4_ and SiO_2_ layers, as such bonds cause absorption losses. However, this step is not compatible with standard photonic foundry processes, especially the complementary metal–oxide–semiconductor (CMOS) process. For the AlGaAsOI waveguide, more than 90% of the mode is confined inside the waveguide core and the cladding loss is negligible.

**Fig. 5:**
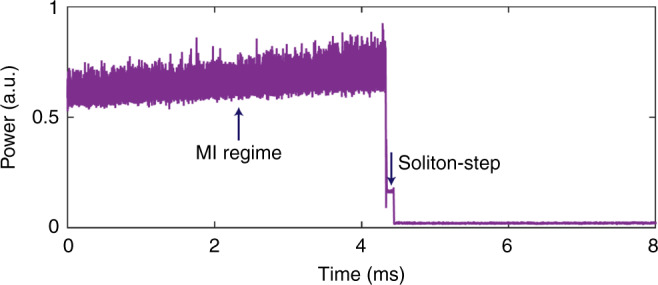
Response of comb power when laser is swept through the AlGaAs resonance. The step-like trace indicates a transition to the soliton state.

To further utilize such efficient frequency-comb generation in the AlGaAsOI platform, the next essential step is to access soliton formation, which enables stable and low-noise states for comb operations^42^. Usually fast frequency scanning of the pump laser is required to trigger the soliton formation in integrated microresonators, due to the fast thermal timescales. When the pump frequency is swept across the resonance, the transition from modulation instability (MI) state to soliton regime leads to a large drop in comb power and therefore a large temperature change, shifting the cavity resonances and making the soliton state unstable and hard to access. As a result, the frequency scanning speed of pump laser needs to be fast for resonators with high absorption and strong thermo-optic effects. For AlGaAsOI resonators, this problem becomes even more challenging than in dielectrics, because AlGaAs’ thermo-optic coefficient (2.3 × 10^−4^ K^−1^) is one order of magnitude higher compared with that of Si_3_N_4_ (2.4 × 10^−5^ K^−1^) or Silica (0.8 × 10^−5^ K^−1^), which leads to a large thermal triangle (see Supplementary Fig. 2). However, thanks to the highly efficient process in this platform, the pump power required for soliton generation is much lower compared with the power in previous experiments, which relieves the thermal effect inside the cavity and therefore the requirement on scanning speed of the laser. Here we observed a step transition from MI to stable comb state during laser scanning, as shown in Fig. 5, which indicates the existence of solitons. The scan speed is only 5 nm s^−1^ and pump power is 2.5 dBm in the bus waveguide for a 450 GHz comb. At this point, due to the relatively large thermal triangle of the resonance, it is challenging to stop the laser frequency at the soliton step and extract the spectrum. Further improvement in the *Q* will reduce the power requirement for soliton generation and facilitate the investigation of soliton formation in this platform.

Recently, several approaches provide other ways to overcome the thermal problem and to generate a low-noise comb state. These include  using an auxiliary laser to provide temperature compensation^43^, or applying dispersion engineering in normal GVD region to eliminate the influence of thermal effect^44^. Further investigation along those directions can improve the potential of the ultra-efficient combs in this platform.

In addition to the microcomb discussed above, the AlGaAsOI platform with ultra-low loss also provides solutions to realize many other nonlinear processes with ultra-high efficiencies on-chip. One example is second order frequency conversion, which is of importance for various applications such as *f*-2*f* self-referencing^27^. Previously, second-harmonic generation (SHG) with record high conversion efficiency based on (Al)GaAsOI waveguides and resonators has been demonstrated based on GaAsOI waveguide^34,45^. Assuming that the six times higher quality factor as demonstrated in this work is applied to the SHG resonators, the efficiency can be improved by a factor of (*Q*_Pump_)^2^*Q*_SHG_, which is more than 200 and in this case is 10^8^ %W^−1^ level. Since AlGaAsOI supports both of the most efficient second- and third-order nonlinear effects in integrated photonics, it holds the potential to simultaneously incorporate self-referencing using SHG together with octave-spanning Kerr comb generation. It should also be noted that in quantum optics, efficient χ^(2)^ and χ^(3)^ processes are important for several processes, e.g., the spontaneous parametric down-conversion^46^ and spontaneous four wave mixing^41^. Moreover, other nonlinear properties of AlGaAs, such as the photoelastic and piezoelectric effects^47^, can be harnessed by utilizing the high-*Q* cavity in optomechanics.

Besides the significance in nonlinear and quantum photonics, this work will also have far-reaching impact to traditional PICs’, both in academia and industry. The AlGaAsOI waveguide structure now enables similar performance compared with a fully etched SOI waveguide^48^, but with much lower nonlinear loss. This makes such a platform suitable for high power and narrow linewidth lasers, optical buffer devices and photonic sensors^49^. Furthermore, compared with SOI, the epi of (Al)GaAs is compatible with the III–V laser gain medium, and therefore can dramatically simplify the current integration technology of heterogeneous III–V on Si, without sacrificing device performance.

In conclusion, we demonstrated a low loss AlGaAsOI platform and applied it to create microcavities with a *Q* factor beyond 1.5 × 10^6^. These high-*Q* devices in combination with the high nonlinearity of the AlGaAsOI waveguides enabled ultra-efficient frequency combs. A record low threshold power around 36 µW for 1 THz comb was achieved. Moreover, pumping the resonator with 300 µW power leads to an efficient comb broadening to a span over 250 nm. The observation of a soliton step in the AlGaAsOI platform has also been reported. This demonstration opens up many opportunities for ultra-efficient nonlinear photonics in this platform. Furthermore, it paves the way for fully integrated nonlinear PICs in the future.

## Methods

### Fabrication

In this work, the single crystalline AlGaAs epi chip is ordered from an outside vendor (Landmark Optoelectronics corporation), whose layer structure from top to bottom is: a [001] orientated 400-nm-thick Al_0.2_Ga_0.8_As film on a 500-nm-thick Al_0.8_Ga_0.2_As layer grown on a 500-µm-thick GaAs substrate. A 5-nm-thick Al_2_O_3_ layer was deposited on the top surface of the epi by atomic layer deposition (ALD) for passivation. This chip was then bonded onto a Si wafer with 3-µm-thick thermal SiO_2_ layer on top after plasma activation. The thermal SiO_2_ layer was prepatterned by inductively coupled plasma (ICP) etching to form cross-shape vertical channels (VCs) with 50 µm spacing and 3 µm depth for gas release. The bonded piece was annealed at 100 °C for 24 h under 1 MPa pressure to enhance the bonding strength. Afterward, mechanical polishing was applied to lap the GaAs substrate thickness down to 70 µm. The remaining GaAs substrate was removed by wet etching with H_2_O_2_:NH_4_OH (30:1) and the Al_0.8_Ga_0.2_As layer was removed by dilute hydrofluoric (∼2.5%) acid.

After substrate removal, 5-nm-thick Al_2_O_3_ layer for passivation and 100 nm SiO_2_ hardmask were deposited on the Al_0.2_Ga_0.8_As thin-film by ALD, respectively. The wafer was then patterned by using photoresist (UV6^TM^-0.8) and deep ultraviolet (DUV) lithography followed. Prior to the photoresist, an anti-reflective (AR) coating (DUV-42P) was used to suppress the backreflection. The photoresist was spun at 5000 rpm with a thickness of ∼600 nm. After the development of photoresist, a thermal reflow treatment was applied at 155 °C for 3 min to smooth the sidewalls of the patterns. The reflow process was done by simply putting the wafer on a hotplate which was already set at 155 °C, and after the treatment the wafer was moved away from the hotplate and cooled in air. ICP etchings with O_2_ and CHF_3_/CF_4_/O_2_ gas chemistries were then applied to etch the AR coating and SiO_2_ hardmask, respectively. Afterward, an ICP etching step with a Cl_2_/N_2_ gas chemistry was applied to etch the Al_0.2_Ga_0.8_As layer. Finally, the sample was passivated by 5-nm-thick Al_2_O_3_ by ALD and then clad with 1.5-µm-thick SiO_2_ by PECVD deposition.

### Frequency-comb characterization

For testing frequency combs, we used ring resonators with 1 THz and 450 GHz FSR, both of whose waveguide cross section geometry is 400 × 700 nm. The chip facets have an inverse taper structure, interfaced with lensed fiber. The coupling loss per facet is around ∼5 dB. The light source used in the experiment is a tunable laser at C band (Keysight 81608A). When measuring the threshold power and broad comb operation, the pump laser is slowly tuned into the resonance (from red detune to blue) and the transmitted pump light and generated comb spectrum are recorded by an optical spectrum analyzer (OSA) (YOKOGAWA AQ6375). For the soliton generation experiment, the laser is automatically swept over the resonance by using laser’s build-in scanning function. The output fiber is connected to a waveshaper (Finisar 4000A), splitting the pump light and the generated other comb lines. The two channels of light are received by two photodetectors (New Focus, Model 1811), respectively, which are monitored by an oscilloscope (Tektronix MSO64) for recording power traces.

## Supplementary information

Supplementary information

## Data Availability

The data that support the findings of this study can be accessed at https://zenodo.org/record/3607042#.X8qgac1Ki70. Additional information is available from the corresponding author upon reasonable request.
