# Author Correction: Ultra-efficient frequency comb generation in AlGaAs-on-insulator microresonators

**DOI:** 10.1038/s41467-021-22031-4

**Published:** 2021-03-16

**Authors:** Lin Chang, Weiqiang Xie, Haowen Shu, Qi-Fan Yang, Boqiang Shen, Andreas Boes, Jon D. Peters, Warren Jin, Chao Xiang, Songtao Liu, Gregory Moille, Su-Peng Yu, Xingjun Wang, Kartik Srinivasan, Scott B. Papp, Kerry Vahala, John E. Bowers

**Affiliations:** 1 Department of Electrical and Computer Engineering, University of California, Santa Barbara, CA 93106 USA; 2 State Key Laboratory of Advanced Optical Communications System and Networks, Peking University, Beijing, 100871 China; 3 T. J. Watson Laboratory of Applied Physics, California Institute of Technology, Pasadena, CA 91125 USA; 4 School of Engineering, RMIT University, Melbourne, VIC 3000 Australia; 5 Microsystems and Nanotechnology Division, National Institute of Standards and Technology, Gaithersburg, MD 20899 USA; 6 Time and Frequency Division, National Institute of Standards and Technology, Boulder, CO 80305 USA

**Keywords:** Microresonators, Nonlinear optics, Frequency combs

Correction to: *Nature Communications* 10.1038/s41467-020-15005-5, published online 12 March 2020.

The original data availability statement for this Article incorrectly listed a link to data file. This has now been corrected to https://zenodo.org/record/3607042#.X8qgac1Ki70 in the PDF and HTML versions of the Article.

The original version of Table 1, numerical value in the third row from bottom and third column was incorrectly listed $7.8 \times 10^{ - 19}$, this has now been corrected to corrected to $6 \times 10^{ - 18}$ in the PDF and HTML versions of the Article.

The original version of the Article in ‘Results', ‘waveguide loss reduction’ sub-section, third sentence, incorrectly used Ref. [2] in the exponent, this has now been corrected to $\left( {n_{{\mathrm{core}}}^2 - n_{{\mathrm{clad}}}^2} \right)$ in the PDF and HTML versions of the Article.

