# Supplementary material for: Ultra-efficient frequency comb generation in AlGaAs-on-insulator microresonators
Source: Nat Commun. 2020 Mar 12;11:1331. doi: 10.1038/s41467-020-15005-5 (PMC7067760; doi:10.1038/s41467-020-15005-5)
Supplement: Supplementary file 1 — Supplementary information [file 41467_2020_15005_MOESM1_ESM.pdf]

Supplementary Information

Ultra-efficient frequency comb generation in AlGaAs-on-insulator microresonators

Lin Chang, Weiqiang Xie, Haowen Shu, Qi-Fan Yang, Boqiang Shen, Andreas Boes, Jon D. Peters, Warren Jin, Chao Xiang, Songtao Liu, Gregory Moille, Su-Peng Yu, Xingjun Wang, Kartik Srinivasan, Scott B. Papp, Kerry Vahala, and John E. Bowers

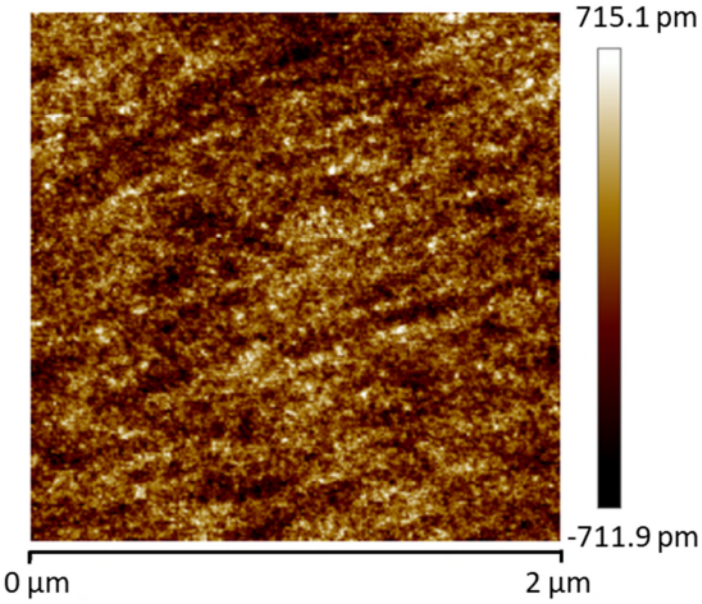

Supplementary Figure 1: Surface roughness characterization. Surface profile of the epi wafer measured by Atomic Force Microscope (AFM). The Root Mean Square (RMS) roughness is ~ 0.2 nm.

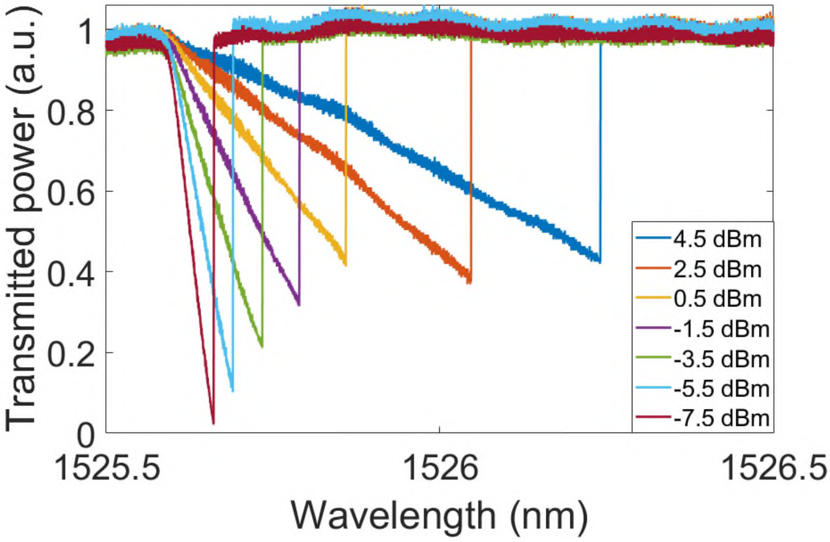

Supplementary Figure 2: Thermal triangle measurement. Measured thermal triangles of a 450 GHz resonator under different on-chip power levels.
